# A Rotary Piezoelectric Electromagnetic Hybrid Energy Harvester

**DOI:** 10.3390/mi16070807

**Published:** 2025-07-11

**Authors:** Zhiyang Yao, Chong Li

**Affiliations:** School of Mechanical Engineering, Jiangsu University of Science and Technology, Zhenjiang 212100, China; 222241802734@stu.just.edu.cn

**Keywords:** energy harvester, rotary motion, output power, planetary gear

## Abstract

To collect the energy generated by rotational motion in the natural environment, a piezoelectric electromagnetic hybrid energy harvester (HEH) based on a planetary gear system is proposed. The harvester combines piezoelectric and electromagnetic effects and is mainly used for collecting low-frequency rotational energy. The HEH has a compact structure and contains four sets of piezoelectric energy harvesters (PEHs) and electromagnetic energy harvesters (EMHs) inside. The working principle of the energy harvester is analyzed, its theoretical model is established, and a simulation analysis is conducted. To verify the effectiveness of the design, an experimental device is constructed. The results indicate that the HEH can generate an average output power of 250 mW under eight magnets and an external excitation frequency of 7 Hz. In actual power supply testing, the HEH can light up 60 LEDs and provide stable power supply for the temperature–humidity meter.

## 1. Introduction

The development of microelectromechanical technology (MENS) and wireless sensor networks (WSNs) has greatly promoted the popularization of the Internet of Things (IoT) and it has been widely applied in fields such as environmental monitoring, smart cities, and industrial equipment health management [1,2,3]. The external power supply problem of sensors has become an obstacle to the sustainable development of IoT technology. However, self-powered technology can effectively solve the above problems [4,5].

There is abundant low-frequency mechanical vibration energy in the natural environment, such as wind energy [6,7,8], wave energy in the ocean [9,10], human motion energy in daily life [11,12,13], and so on, with most of the energy frequencies below 100 Hz. The current mainstream mechanisms for converting other forms of energy into electrical energy include piezoelectric [14,15,16], electromagnetic [17,18,19], magnetic energy, triboelectricity [20,21,22], and electrostatic mechanisms. Among them, the piezoelectric energy harvester utilizes the piezoelectric effect to convert mechanical vibration energy into electrical energy, and has the characteristics of a high energy density, no electromagnetic interference, and compact structure. The electromagnetic type is based on Faraday’s law of electromagnetic induction, which uses a coil to generate induced current in a changing magnetic field, especially suitable for low-frequency and large-displacement-vibration environments. These two forms of energy harvesting are considered more suitable for harvesting energy in mechanical vibrations due to their high conversion efficiency and resistance to environmental influences.

Liao et al. [23] proposed a centrifugal spring mechanism that could achieve adaptive magnetic force enhancement at different wind speeds. This structure has the characteristics of an ultra-low start-up wind speed (1.5 m/s), high output power (5.97 mW at 4.75 m/s), and a wide bandwidth (1.5–5.5 m/s), while demonstrating the dual functions of powering microelectronics and implementing intelligent wind sensing through a CNN algorithm. To achieve the kinetic energy recovery of complex ship movements, Li et al. [24] developed a multiple-degree-of-freedom energy harvesting system based on a club mechanism. The device achieved a maximum output power of 173.1 μW in actual ship testing and demonstrated efficient capacitor charging capability, providing a self-powered solution for low-power equipment on board ships. Yin et al. [25] proposed a rolling-swing electromagnetic energy harvester that utilized the reverse rotation between a magnet and a coil to amplify magnetic field changes under ultra-low frequency vibrations (0.1–3 Hz). The energy harvester adopted a unique alternating magnetic pole arrangement to double the output voltage, achieving a milliwatt-level power output, and successfully demonstrating the ability to power multiple low-power devices (such as thermometers and calculators) under laboratory and actual walking conditions, suitable for self-powered IoT applications.

Due to the randomness and multi-directional dispersion of vibrations in natural environments, a single energy harvesting mechanism often can only collect energy in a specific direction, and its output form is relatively limited [26,27,28]. Taking piezoelectric and electromagnetic mechanisms as examples, the former can output a high voltage but has a limited output current, while the latter outputs a low voltage but a high current. Therefore, some researchers have designed composite-vibration energy harvesting devices by integrating two or more forms of energy harvesting [29,30,31] to improve overall energy harvesting efficiency. Zhao et al. [32] proposed an intelligent mechanical wave energy harvester. That design adaptively converted low-frequency, irregular wave motion into reciprocating rolling and high-speed unidirectional rotation through a ratchet and magnetic coupling. The prototype could operate effectively at ultra-low frequencies (0.1 Hz), charging a 0.47 F capacitor to 5 V within 6 min under simulated waves, achieving a self-powered wireless marine environment monitoring system for sensing light, temperature, and water quality. Bai et al. [33] proposed a friction piezoelectric electromagnetic hybrid wind energy collector based on a bouncing bistable mechanism. This design integrated three energy conversion mechanisms at the maximum strain, contact area, and displacement point, achieving a low wind speed (3 m/s), high power density, and continuous stable operation. It successfully lit up 1000 LED lights and demonstrated self-powered wireless environmental monitoring (such as temperature and wind speed). In addition, Zhao et al. [34] designed a high-performance frictional electromagnetic hybrid nanogenerator based on a NdFeB/EC composite film for collecting wind energy. At a wind speed of 15.5 m/s, their nanogenerator unit could generate an open-circuit voltage of 55 V, a short-circuit current of 5.4 μA, and a maximum power of 99.2 μW. The electromagnetic generator unit generated an open-circuit voltage and a short-circuit current of approximately 0.16 V and 5.93 μA, respectively, with an output power of 2.5 μW.

This article proposes a piezoelectric electromagnetic hybrid energy harvester (HEH) based on a planetary gear system design, aimed at efficiently capturing low-frequency mechanical energy in the environment. The innovation of this device lies in the gear meshing transmission through a planetary gear system (transmission ratio 2:1), which amplifies the external excitation frequency twofold to achieve a higher energy output. The device has a compact structure and contains four sets of piezoelectric energy harvesting units (PEHs) and four sets of electromagnetic energy harvesting units (EMHs) inside. In addition, the device also adopts a center crank slider mechanism to convert the external rotational excitation into a linear motion of magnets. The movement of the magnet drives the cantilever beam to bend and deform, thereby exciting the PZT to output electrical energy. On the other hand, the change in magnetic flux inside the coil generates an induced electromotive force, which outputs the corresponding induced current. This magnetic coupling excitation design improves the service life and reliability of the HEH.

## 2. Design and Operating Principle

**Figure 1:**
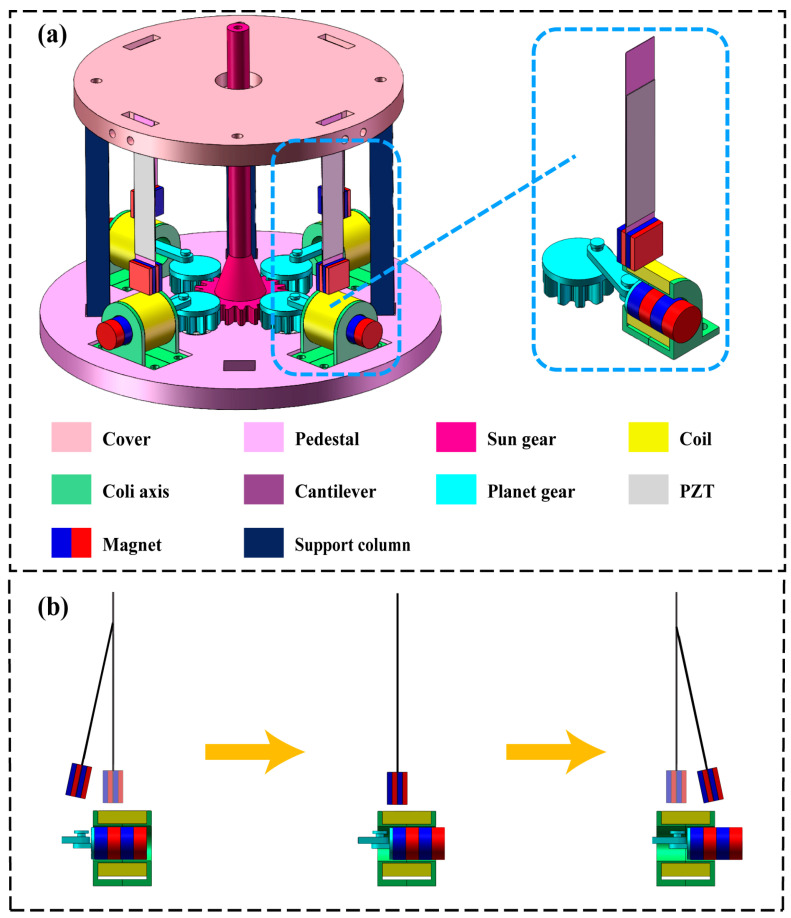
The piezoelectric electromagnetic hybrid energy harvester. (**a**) Schematic diagram of the prototype structure; (**b**) the changing process of the cantilever during work.

The hybrid energy harvester (HEH) proposed in this article consists of four circularly distributed piezoelectric energy harvesters (PEHs) and four circularly distributed electromagnetic energy harvesters (EMHs), as shown in Figure 1.

The EMH is mainly composed of circular magnets, coils, and coil tubes. By the reciprocating motion of a circular magnet inside the tube, a constantly changing magnetic field is generated around the coil, resulting in induced current inside the coil. The PEH is mainly composed of a PZT material, cantilever beam, and rectangular magnet attached to the bottom of the cantilever beam. Due to the constantly changing gravitational force between the circular magnet and the square magnet, the cantilever beam deforms, causing voltage to be generated inside the PZT material. The HEH utilizes magnetic coupling mechanisms to achieve the conversion of mechanical energy into electrical energy. Table 1 and Table 2 present in detail the structural dimensions of the HEH and the technical parameters of its main components. The size of the entire HEH is Φ140 × 95 mm. Due to its compact size and low installation space requirements, the device has good application prospects.

When the sun gear of the HEH is excited by external rotational energy, it drives the rotation of four planet gears through gear meshing. At the same time, a centering crank slider mechanism is attached to the planet gear, with one end of the connecting rod connected to a circular magnet, converting the rotational motion of the planet gear into the linear reciprocating motion of the magnet. Figure 1b illustrates the working principles of the EMH and PEH. On the one hand, the linear reciprocating motion of the magnet causes the magnetic induction intensity around the coil to constantly change, thereby continuously generating the current induced in the coil. On the other hand, circular magnets attract square magnets to move synchronously during their motion, causing deformation of the cantilever beam and generating a polarization voltage inside the PZT material, achieving the output of piezoelectric energy. Throughout the entire motion process, due to the constant existence of the magnetic coupling, the HEH can achieve a stable and efficient energy output. The characteristic of this article is that by adjusting the gear ratio of the sun gear and the planet gear, the external low-frequency energy excitation can be amplified, thereby effectively achieving the collection of low-frequency energy. The transmission ratio of this HEH is 2:1, which can amplify the external excitation applied to the sun gear twofold and transmit it to the EMH and PEH, thereby achieving a greater energy output.

## 3. Modelling and Analysis

### 3.1. Theoretical Modeling

**Figure 2:**
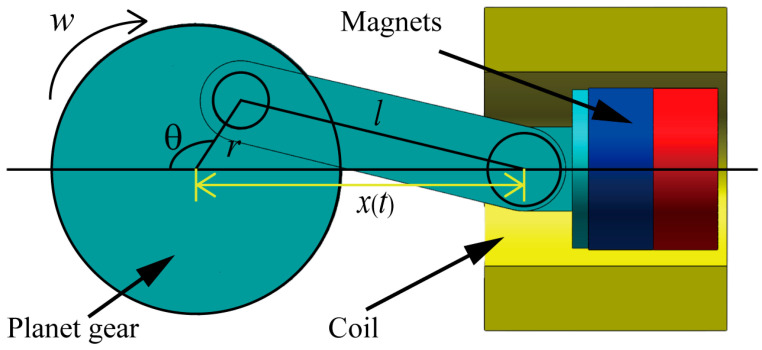
Schematic diagram of the movement of the magnets.

The HEH designed in this article utilizes the centric slider crank mechanism to convert the rotational motion of the planet gear into the linear motion of a permanent magnet. The motion structure is shown in Figure 2.

Assuming that the angular velocity of the planet gear during rotation is ω, the time is *t*, and the initial state is θ = 0°, the displacement *x*(*t*) of the permanent magnet is:

(1) $$x(t)=rcos(\omega t)+\sqrt{l^{2}-r^{2}sin^{2}(\omega t)},$$

where *r* is the distance radius from the planet gear to the connecting rod, and *l* is the length of the connecting rod.

Meanwhile, by taking the derivative of *x*(*t*), the moving speed *v*(*t*) of the permanent magnet can be calculated as follows:

(2) $$\begin{array}{cc} v(t) & =\frac{dx}{dt}\;=-r\omega sin(\omega t)-\frac{r^{2}\omega sin(\omega t)cos(\omega t)}{\sqrt{l^{2}-r^{2}sin^{2}(\omega t)}}\\ & =-r\omega sin(\omega t)\left[1+\frac{\;rcos(\omega t)}{\sqrt{l^{2}-r^{2}sin^{2}(\omega t)}}\right] \end{array}.$$

The induced electromotive force generated by the law of electromagnetic induction is divided into an induced electromotive force and a dynamic electromotive force. In the EMH system designed in this article, the magnetic field around the coil constantly changes due to the motion of the permanent magnet, so the electromotive force generated inside the coil is an induced electromotive force. The electromotive force can be expressed as:

(3) $$\begin{array}{ll} E_{1} & =-\frac{d\Phi }{dt}\\ & =NAv(t)\frac{dB}{d\xi } \end{array},$$

where *N* and *A* are the number of turns and cross-sectional area of the coil; *B* is the magnetic induction strength of the magnet, and *ξ* is the instantaneous distance between the center *x*1 of the coil and the center *x*(*t*) of the permanent magnet, $\xi =x_{1}-x(t)$.

The relationship between the magnetic induction intensity generated by a magnet and the instantaneous distance between the coil and the magnet is as follows:

(4) $$B(\xi )=\frac{B_{r}}{2}\left[\frac{\xi +\frac{L}{2}}{\sqrt{{\left(\xi +\frac{L}{2}\right)}^{2}+R^{2}}}-\frac{\xi -\frac{L}{2}}{\sqrt{{\left(\xi -\frac{L}{2}\right)}^{2}+R^{2}}}\right],$$

where *B_r_* is the residual magnetic flux density of the permanent magnet itself, and *R* and *L* are the radius and length of the cylindrical magnet, respectively.

Due to the constant motion of permanent magnets, the magnetic field strength around the coil is also constantly changing, and the magnetic field gradient can be used to represent the rate of change in magnetic induction intensity around the coil with the instantaneous distance, expressed as:

(5) $$\frac{dB}{d\xi }=\frac{B_{r}R^{2}}{2}\left(\frac{1}{{\left[{\left(\xi +\frac{L}{2}\right)}^{2}+R^{2}\right]}^{3/2}}-\frac{1}{{\left[{\left(\xi -\frac{L}{2}\right)}^{2}+R^{2}\right]}^{3/2}}\right).$$

Therefore, the induced electromotive force generated by the final EMH can be expressed as:

(6) $$\begin{array}{l} E_{1}=-NA\frac{B_{r}R^{2}}{2}r\omega sin(\omega t)\left[1+\frac{\;rcos(\omega t)}{\sqrt{l^{2}-r^{2}sin^{2}(\omega t)}}\right]\\ \times \;\left(\frac{1}{{\left[{\left((x_{c}-x_{m}(t))+\frac{L}{2}\right)}^{2}+R^{2}\right]}^{3/2}}-\frac{1}{{\left[{\left((x_{c}-x_{m}(t))-\frac{L}{2}\right)}^{2}+R^{2}\right]}^{3/2}}\right) \end{array}.$$

**Figure 3:**
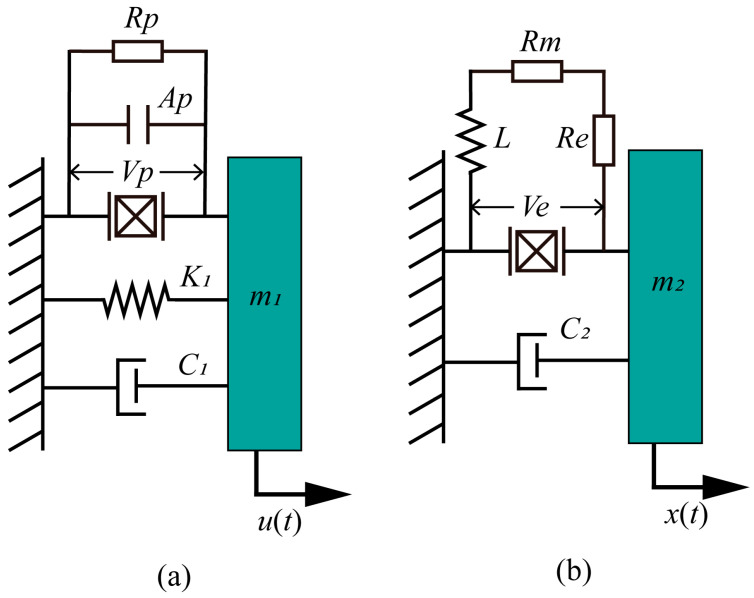
Equivalent model of the energy harvester. (**a**) Piezoelectric module; (**b**) electromagnetic module.

To better understand the working principle of the HEH structure, an equivalent dynamic model was established, as shown in Figure 3. The equivalent models of the PEH and EMH are shown in Figure 3a and 3b, respectively.

In the equivalent model of the PEH shown in Figure 3, *R_p_* and *A_p_* represent the load resistance and equivalent capacitance in the piezoelectric system circuit; *V_p_* represents the output voltage of the piezoelectric system; *K*_1_ is the equivalent stiffness of the cantilever beam; *C*_1_ is the mechanical piezoelectric equivalent damping coefficient, consisting of a piezoelectric damping coefficient and a mechanical damping coefficient; *M*_1_ is the equivalent mass of the cantilever beam and its end magnets; *U*(*t*) represents the external motivation it receives. For the equivalent model of the EMH, *R_m_* and *R_e_* represent the load resistance and internal resistance in the electromagnetic system circuit, and *L* represents the equivalent inductance of the electromagnetic system; *V_e_* represents the output voltage of the electromagnetic system; *C*_2_ is its equivalent damping coefficient, mainly derived from air resistance; *M*_2_ is the equivalent mass of the magnet inside the coil tube; and *x*(*t*) represents the external excitation received by the electromagnetic system.

According to the equivalent dynamic model of the device, the motion’s differential equation of the piezoelectric energy harvesting part can be established as follows:

(7) $$m_{1}ü(t)+C_{1}\dot{u}(t)+K_{1}u(t)=F_{m}-\gamma_{1}V_{0}(t),$$

where *γ*_1_ is the electromechanical coupling coefficient of the PEH, and *F_m_* is the magnetic force between magnets, which can be expressed as:

(8) $$F_{m}=\frac{\mu_{0}\nu_{1}\nu_{2}J_{1}J_{2}}{4\pi }\left(\frac{5p}{{\left(p^{2}+R_{0}^{2}\right)}^{\frac{5}{2}}}-\frac{5p^{3}}{{\left(p^{2}+R_{0}^{2}\right)}^{\frac{7}{2}}}\right),$$

where *μ*_0_ is the magnetic permeability of the vacuum medium, *ε*_1_ is the volume of the magnet inside the coil tube, *ε*_2_ is the volume of the response magnet at the end of the piezoelectric cantilever beam, *J*_1_ is the magnetization intensity of the magnet inside the coil tube, *J*_2_ is the magnetization intensity of the response magnet at the end of the piezoelectric cantilever beam, *R*_0_ is the distance between the center of the driving magnet at the end of the piezoelectric cantilever beam and the magnet inside the coil tube, and *p* is the distance between the magnet inside the coil tube and the coil tube.

From Equation (8), it can be inferred that the magnetic force Fm is not only related to the distance between magnets but also to the number of magnets and the properties of the magnets themselves.

The PEH in the device can be equivalent to a parallel connection of voltage source *V_p_*, capacitor *A_p_*, and resistor *R_p_*. According to the equivalent power model and Kirchhoff’s law, the following can be obtained:

(9) $$A_{p}{\dot{V}}_{p}(t)+V_{p}(t)R_{p}^{-1}-\gamma_{1}\dot{u}(t)=0.$$

When the piezoelectric cantilever beam is excited by a magnetic force, according to the analysis of the positive piezoelectric effect, the open-circuit voltage output of the PEH can be obtained as:

(10) $$V_{p}=\frac{3d_{31}t_{h}Et_{b}\sigma F_{m}}{8m_{1}t_{l}^{2}A_{p}\omega^{2}},$$

where *t_b_* is the thickness of the piezoelectric sheet, *t_h_* is the height of the piezoelectric cantilever beam, *t_l_* is the length of the piezoelectric cantilever beam, *σ* is the average surface stress of the piezoelectric cantilever, *E_p_* is the Young’s modulus of the piezoelectric cantilever beam, and *ω* is the excited vibration frequency.

Furthermore, the output power of the PEH can be derived as:

(11) $$P_{1}=\frac{V_{p}^{2}}{R_{p}}=\frac{9}{64}\left[\frac{{\left(d_{31}t_{h}E_{p}t_{b}\sigma F_{m}\right)}^{2}}{R_{p}m_{1}^{2}t_{l}^{4}C_{pe}^{2}\omega^{4}}\right].$$

According to the established equivalent model, the motion’s differential equation of the electromagnetic energy harvesting part can be established, and its expression is:

(12) $$m_{2}\ddot{x}(t)+C_{2}\dot{x}(t)=F_{m}-\rho_{1}I_{0}(t),$$

where *I*_0_(*t*) is the voltage across the external load resistor of the electromagnetic part, and *ρ*_1_ is the electromechanical coupling coefficient of the electromagnetic energy harvesting part. When the magnet inside the coil tube moves in a straight line, the coil cuts the magnetic induction line, and a corresponding induced electromotive force is generated inside, which can be expressed as:

(13) $$V_{e}=\frac{d\phi }{dt}=nB_{0}L_{0}\dot{x}(t),$$

where *n* is the number of coil turns, *B*_0_ is the magnetic induction intensity, and *L*_0_ is the effective length of the coil cutting the magnetic induction line.

In the equivalent circuit of the electromagnetic part, since the inductance of the coil is very small and can be ignored, only the load’s internal resistance *R_m_* and the internal resistance *R_e_* of the coil are considered. Therefore, the output power of the coil part can be expressed as:

(14) $$P_{2}=\frac{1}{T}\int_{0}^{T}\frac{V_{e}^{2}}{R_{m}+R_{e}}dt=\frac{1}{2}\frac{{\left(nB_{0}L_{0}\right)}^{2}}{R_{m}+R_{e}}\omega^{2}z^{2},$$

where *ω* represents the excitation frequency, and *z* represents the displacement of the magnet relative to the coil.

By combining Equations (11) and (14), the total output power of the entire device can be obtained as follows:

(15) $$P=P_{1}+P_{2}=\frac{9}{64}\left[\frac{{\left(d_{31}t_{h}E_{p}t_{b}\sigma F_{m}\right)}^{2}}{R_{p}m_{1}^{2}t_{l}^{4}C_{pe}^{2}\omega^{4}}\right]+\frac{1}{2}\frac{{\left(nB_{0}L_{0}\right)}^{2}}{R_{m}+R_{e}}\omega^{2}z^{2}$$

### 3.2. Simulation Analysis

To verify the output characteristics and force conditions of the HEH, this paper used COMSOL 6.2 software and ANSYS 2022R1 software to simulate the HEH and verify the effectiveness of the model device and mathematical model.

The EMH module consisted of N52 permanent magnets and induction coils. Due to the symmetrical structure of the EMH, a two-dimensional plane was chosen for modeling and simulation. In the parameter setting section, the number of turns of the coil in the model was 432, the number of magnetic oscillators was 8, the magnetic induction intensity was 1.48 T, the peak displacement was set to 5 mm, and the excitation method was simplified to a sine wave. This model was mainly used to solve the electrical output characteristics of coils, so the field integration method was used for the calculation.

**Figure 4:**
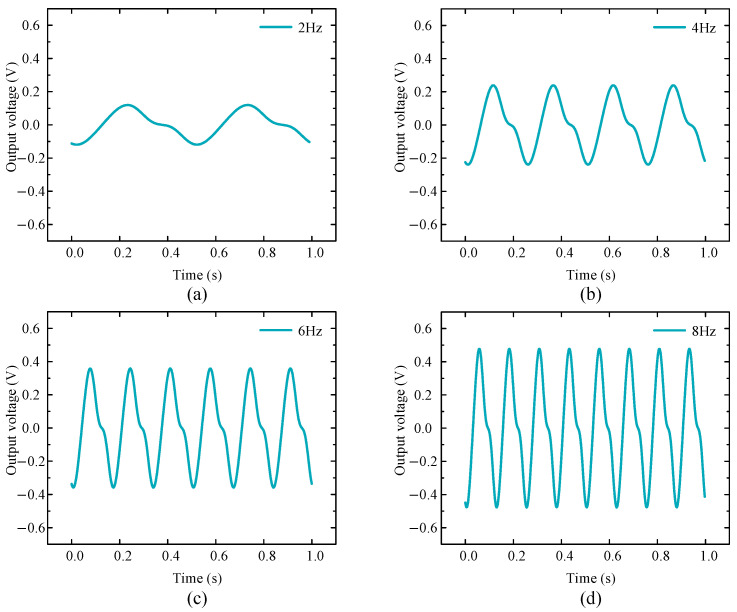
Output voltage of the electromagnetic module under different frequencies: (**a**) 2 Hz; (**b**) 4 Hz; (**c**) 6 Hz; (**d**) 8 Hz.

Figure 4 shows the voltage changes of the EMH under four different excitation frequencies. From the results, as the excitation frequency increased, the motion period gradually shortened, while the peak voltage output produced by the EMH gradually increased, allowing for more energy to be obtained per unit time. In addition to the voltage output characteristics of the EMH, similar conclusions could also be drawn regarding changes in output power.

**Figure 5:**
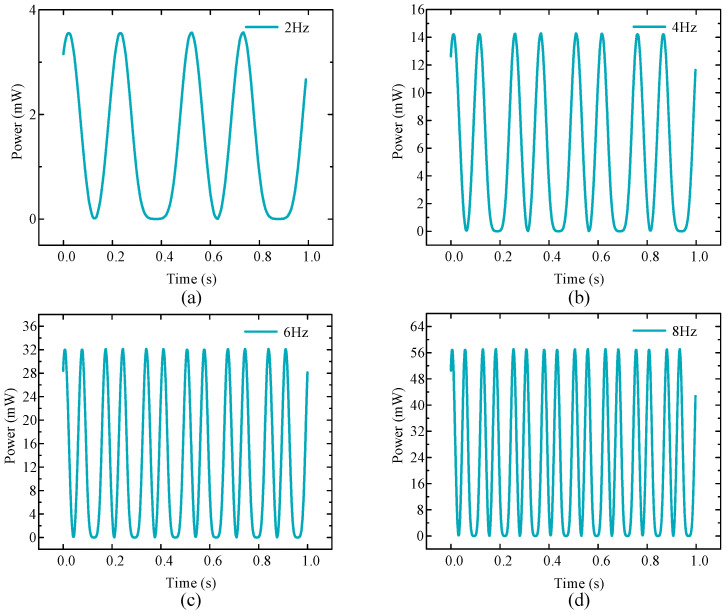
Output power of the electromagnetic module under different frequencies: (**a**) 2 Hz; (**b**) 4 Hz; (**c**) 6 Hz; (**d**) 8 Hz.

Figure 5 shows the output power of the EMH module under different excitation frequencies. As shown in the figure, the output power of the coil increased with the increase in frequency, and the motion period gradually decreased, enabling the EMH to achieve a larger and faster power output per unit time. When the driving frequency was 8 Hz, the maximum output power of the electromagnetic module reached 58 mW.

**Figure 6:**
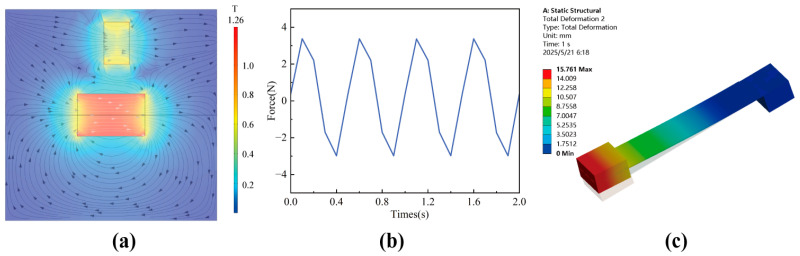
Simulation analysis of the piezoelectric cantilever beam under the action of a magnetic field. (**a**) Magnetic flux density mode; (**b**) force between magnets with time; (**c**) simulated displacement.

Due to the non-contact magnetic excitation used for the vibration excitation of piezoelectric cantilever beams, it is particularly important to analyze the magnetic field and force of the interaction between magnets. Based on the relevant parameters in Table 1 and Table 2, a magnet simulation model was established in COMSOL 6.2 software. The circular magnet was set to a sinusoidal linear reciprocating motion with a peak displacement of 5 mm. Under these conditions, the magnetic field and magnetic force were solved. The magnetic flux density mode between magnets is shown in Figure 6a, with the maximum magnetic field strength near the magnets.

Figure 6b shows the variation in the interaction force between magnets over time. Due to the sinusoidal motion of the circular magnet, its force was approximately expressed as a sine wave, with a maximum value of about 3.3 N and an effective value of 2.3 N. Subsequently, a simulation model of a piezoelectric cantilever beam was constructed in ANSYS 2022R1 software, with one end of the cantilever beam set as a fixed constraint and a pressure of 2.3 N applied to the surface of the bottom magnet to simulate the force situation of the cantilever beam during the motion process. The simulation results showed that the maximum deformation of the piezoelectric cantilever beam under that condition reached 15.761 mm, and this deformation was mainly distributed in the end magnets, which would not cause damage to the PZT. This result indicates that magnetic excitation can effectively deform piezoelectric cantilever beams.

## 4. Experimental Results and Discussion

### 4.1. Output Characteristics Analysis

**Figure 7:**
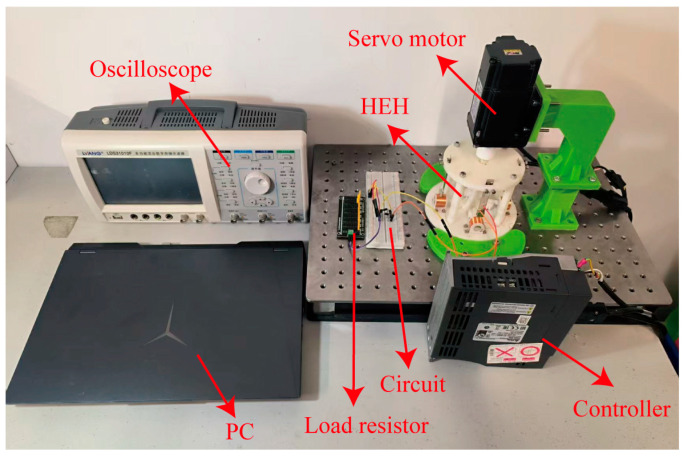
The experimental setup of the HEH.

To further verify the energy output characteristics of the HEH structure under rotational excitation conditions, a prototype of the HEH was manufactured using 3D printing technology, and an experimental setup was established, as shown in Figure 7. We fixed the experimental device on the experimental platform with bolts and fixed the sun gear and planet gear on the pedestal with bearing bolts. We used a servo motor (ASDA-B2) and its driver to drive the sun gear to simulate rotational excitation conditions, the servo motor was connected to the sun gear through a coupling. We used an oscilloscope to display and record the electrical output characteristics of the HEH. In the experiment, the PZT was attached to a cantilever beam using epoxy resin, while the circular magnet was composed of several N52 permanent magnets with a diameter of 10 mm and a height of 2 mm. Considering the low-frequency operating characteristics of the energy harvester, the operating frequency of the servo motor was set to 1–10 Hz by setting parameters on the PC host to control the driver, and the excitation provided by the servo motor was sinusoidal.

**Figure 8:**
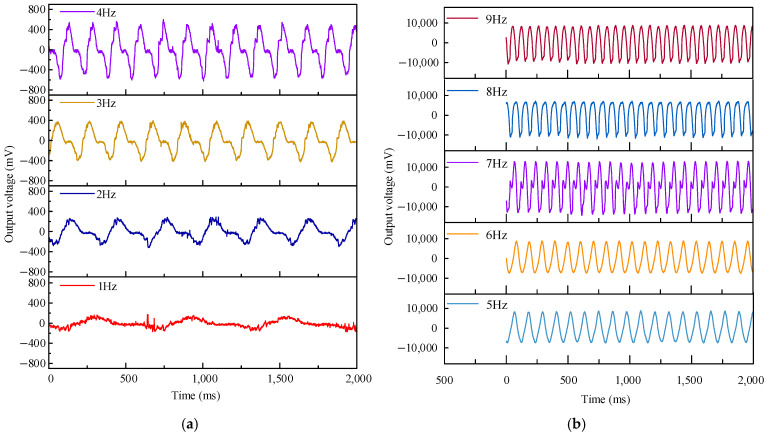
Output voltage of the EMH and PEH under different frequencies. (**a**) Output voltage of the EMH; (**b**) output voltage of the PEH.

To verify the amplification effect of the planetary gear system on an external low-frequency energy excitation, the output voltages of the EMH and PEH in the experimental device were collected, and the results are shown in Figure 8. The frequencies shown in the figure are the excitation frequencies applied by the servo motor to the experimental device. The number of N52 permanent magnets used was eight, and the overall height of the magnets was 16 mm, which was consistent with the parameters of the coil simulation. The waveform of the PEH indicated that 7 Hz was the resonant frequency of the cantilever beam at which the maximum voltage was collected.

Results showed that the experimental waveforms, peak voltages, and periods at each frequency were similar to the simulation results corresponding to twice the frequency in Figure 4. For example, in the experiment, the peak voltage at the 4 Hz excitation frequency was 550 mV with a period of 150 ms, which was consistent with the 500 mV peak voltage and 125 ms period at 8 Hz in the simulation. These results effectively demonstrate that planetary gear systems have a certain amplification effect on external low-frequency energy excitation. However, there was still a deviation between experimental data and simulation data, which may have been mainly due to the friction between the magnet and the coil axis during its motion. This friction hindered the speed at which the coil cuts the magnetic induction line, resulting in a delay in the experimental period compared to the simulation period.

**Figure 9:**
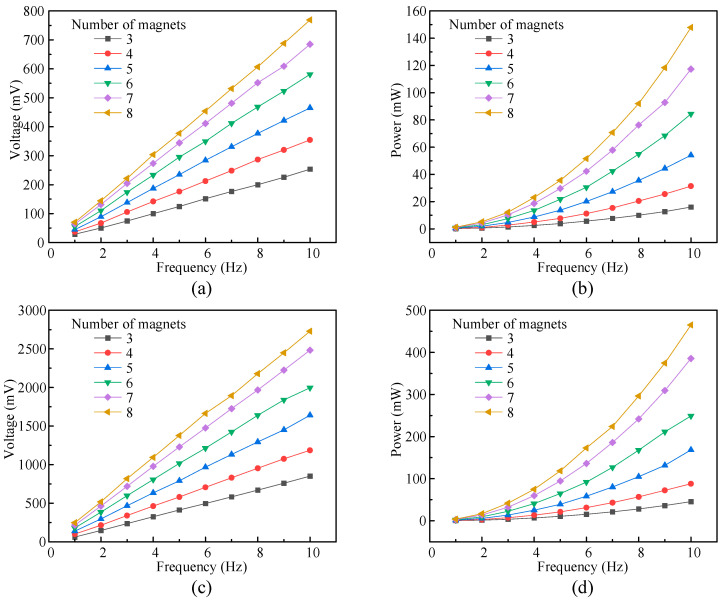
Experimental output results of the EMH. (**a**) Output voltage of a single EMH module; (**b**) output power of a single EMH module; (**c**) output voltage of a multiple-EMH module; (**d**) Output power of a multiple-EMH module.

The number of magnets at the center of the coil also affects the performance of the proposed EMH. Therefore, experimental tests were conducted on the EMH under different quantities of permanent magnets. The effective values of its output voltage were collected using an oscilloscope, and the output frequency of the EMH was calculated. The results are shown in Figure 9.

Figure 9a shows the variation in output voltage of a single EMH group with an excitation frequency for different numbers of permanent magnets. As the excitation frequency and number of magnets increase, the output voltage of a single EMH group also increases accordingly. This is because as the excitation frequency and the number of magnets increase, the magnetic induction intensity around the coil increases, and the speed at which the coil cuts the magnetic induction line accelerates, resulting in an increase in the rate of change in the magnetic flux inside the coil. The combined effect of these factors increases the induced electromotive force generated. Correspondingly, the output power of the EMH also increases, as shown in Figure 9b.

To measure the output characteristics of multiple sets of EMHs in the HEH, four sets of EMHs were connected in series to collect data on their output voltage and power. The results are shown in Figure 9c,d. Under the excitation frequency conditions of eight magnets and 10 Hz, the output voltage and power of the entire EMH could reach 2.728 V and 465 mW. However, the output voltage of the multiple-EMH module was not exactly equal to four times the output voltage of a single EMH. The reason for this may be that the speed or movement path of the four sets of permanent magnets was unstable during the motion process, and there were differences in the rate of change in magnetic flux generated by each set of coils, resulting in phase differences in the induced electromotive force generated by each coil, and the total voltage may have experienced vector superposition due to phase differences.

**Figure 10:**
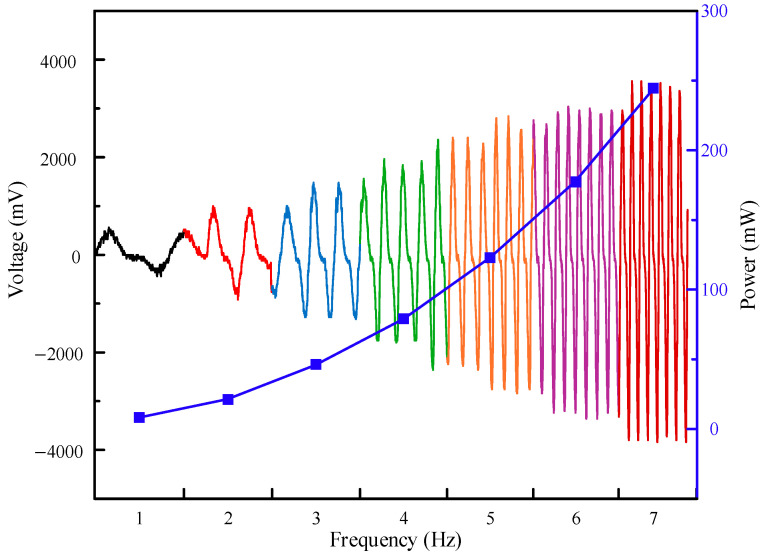
Voltage changes in the EMH during frequency fluctuations.

Figure 10 shows the output voltage of the EMH when the excitation frequency fluctuates. The results show that when the frequency fluctuates, the instantaneous output power changes. In order to achieve a stable output of energy, the collected energy needs to be stabilized, processed, and stored in a battery so that it can supply power to low-power appliances.

**Figure 11:**
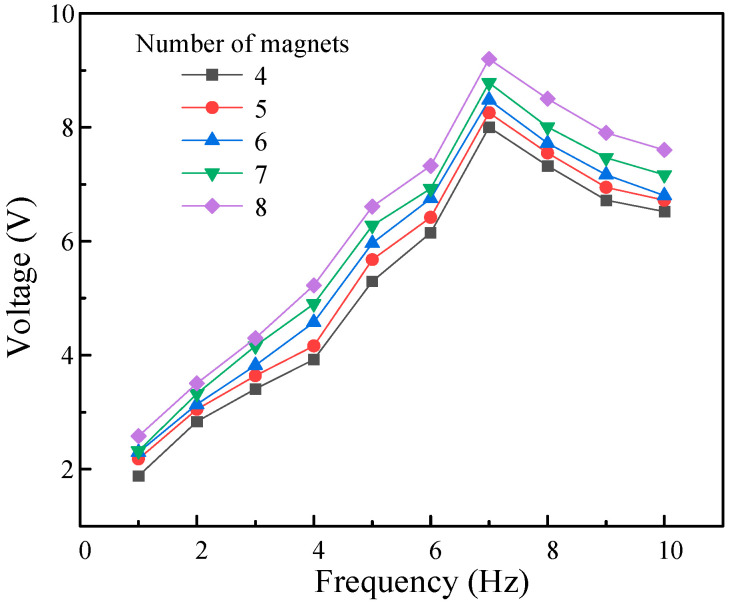
Output voltage of PEH under different numbers of magnets.

For the PEH modules, their electrical output characteristics largely depend on the resonance of the device structure, and the vibration frequency of the cantilever beam is a key external factor affecting the performance of the PEH. This article tested the variation in open circuit voltage with the excitation frequency of the PEH under different numbers of permanent magnets on a self-built experimental device, as shown in Figure 11.

When the number of permanent magnets at the center of the coil was fixed, as the excitation frequency increased from 1 Hz to 10 Hz, the measured output voltage of the PEH showed a trend of first increasing and then decreasing. When the rotational excitation frequency was 7 Hz, the output voltage reached its peak, indicating that the cantilever beam in the PEH was in a resonant state. Since the HEH designed in this article could amplify the external excitation frequency twofold through the planetary gear, when the external excitation frequency reached 7 Hz, the vibration frequency of the cantilever beam in the PEH was 14 Hz, which was highly consistent with the finite element simulation results. The experimental results showed that when configuring eight permanent magnets and using 7 Hz as the excitation frequency, the output voltage of a single PEH module could reach 9.2 V.

**Figure 12:**
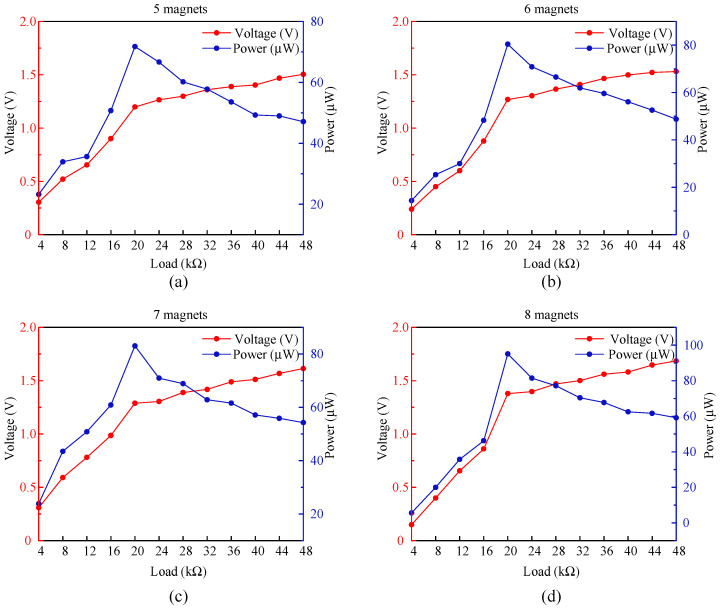
Output voltage and power of the PEH with various loads under different numbers of magnets: (**a**) 5 magnets; (**b**) 6 magnets; (**c**) 7 magnets; (**d**) 8 magnets.

Due to the capacitive properties exhibited by the piezoelectric effect, the internal impedance of the PZT piezoelectric ceramic sheet in the PEH structure is not constant but varies with the external excitation, which directly affects the output voltage and power of the PEH. Therefore, it is crucial to find the best load impedance for the PZT in the operating range. Considering that 7 Hz was the optimum excitation frequency for the PEH structure and the internal impedance of the PZT was not affected by the variation in the structural device’s own parameters, in this paper, the optimal impedance of the PZT was chosen to be estimated at a 7 Hz excitation frequency. A variable load resistor was connected to the output port of the PEH, and an oscilloscope was used to record the output voltage and power of the PEH for different numbers of permanent magnets; the results are shown in Figure 12.

In Figure 12, with the same number of permanent magnets, the output voltage gradually increases with the increase in load resistance, while the output power shows a trend of increasing and then decreasing. Through a comparative analysis, it was found that PEH reached its maximum output power at a 20 kΩ load for all different configurations of the number of permanent magnets. Therefore, 20 kΩ could be considered as the optimum load impedance in this frequency band. Under this load condition, the maximum output power of the PEH module was 95 μW.

Under the external excitation of eight magnets at 7 Hz, the output voltage obtained by experimentally measuring the four EMHs in series was 1.9 V, and the actual output power was calculated to be about 230 mW, so the output efficiency of the actual EMHs was calculated to be 64%.

Similarly, with eight magnets, an external excitation frequency of 7 Hz, and an external load of 20 kΩ, the actual measured output power of the four PEHs was about 180 μW, and the calculated output efficiency of the PEHs was about 47%.

In summary, it can be deduced that the total output power of the HEH reached 250 mW with an overall efficiency of 67% under eight magnets and a 7 Hz external excitation frequency. The loss of efficiency was due to the fact that during the movement of the magnet, its speed or path was not consistent, resulting in a slight difference in the amount of flux generated by each group of coils, which caused a phase difference between the induced electromotive force generated by each coil. This phase difference caused the total output power to be reduced due to vector superposition.

### 4.2. Application Analysis

**Figure 13:**
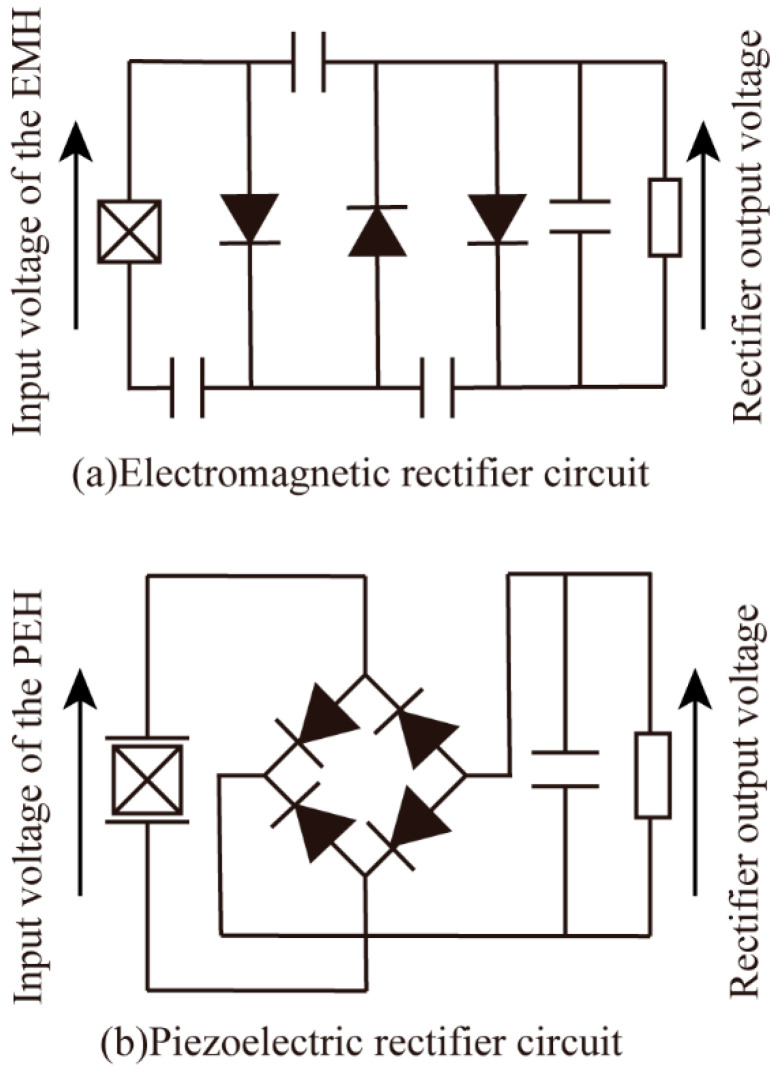
Rectifier circuit of the HEH. (**a**) Electromagnetic rectifier circuit; (**b**) piezoelectric rectifier circuit.

The HEH proposed in this paper is essentially a piezoelectric–electromagnetic hybrid energy harvester. Due to the significant differences in the output electrical characteristics of the EMH and PEH, two independent channels of energy harvesting were used to fully utilize their respective advantages. The electrical energy generated by an EMH has low-voltage and high-current characteristics, so a voltage doubler rectifier circuit, which is a circuit that stores energy through capacitors to realize multiple voltage rectification, was used, as shown in Figure 13a. For the PEH, the electrical energy generated is an alternating current (AC), characterized by a high voltage and low current, which makes it impossible to supply power to the load continuously. For this reason, an AC–DC bridge rectifier circuit was used to efficiently collect the energy generated by the PEH, which consisted of an AC–DC rectifier module and a filter capacitor, as shown in Figure 13b.

By connecting the two rectifier circuits in series, the overall energy harvesting of the HEH was realized, and thus continuous power supply to the load was achieved. Through experiments as well as calculations, under the condition of eight magnets and an external excitation frequency of 7 Hz, the output voltage of the HEH could reach 10.4 V, and the average output power was 250 mW. The results show that the HEH was good at low-frequency energy harvesting.

**Figure 14:**
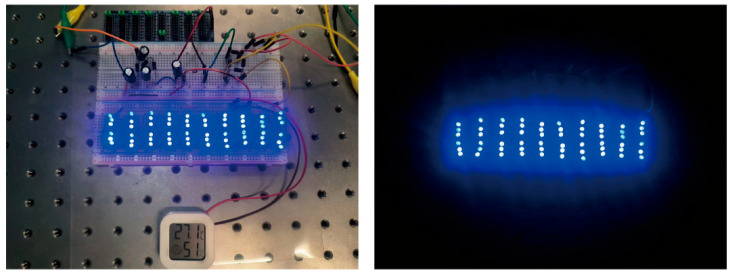
Practical application of the HEH.

To verify the performance of the designed HEH in supplying power to low-power electronic devices, electronic load experiments were conducted. Under eight permanent magnets and a 7 Hz excitation frequency, the HEH successfully lit 60 LEDs and provided a stable power supply for the temperature–humidity meter, as shown in Figure 14. This experimental result indicates that the HEH designed in this paper has the potential to provide reliable energy for some low-power electronic products in low-frequency environments.

**Figure 15:**
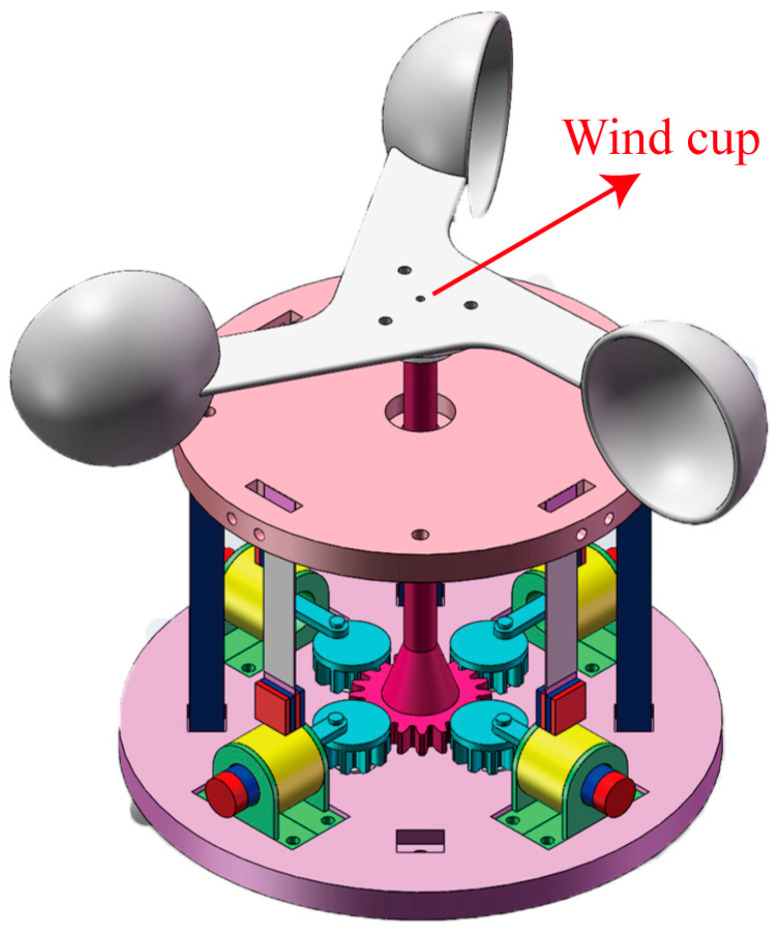
Further practical application of the HEH.

In addition to the experiments mentioned above, further applications for the HEH are possible. In practical applications, the HEH can be combined with wind cups to convert the driving mode from electric motors to wind power, thereby converting wind energy in the natural environment into electrical energy, as shown in Figure 15. At the same time, the rectification circuit of the HEH can be connected to an energy storage circuit for storing the collected electrical energy, which can be used for some small electronic devices such as thermometers, indicator lights, street lighting, etc.

## 5. Conclusions

This article proposed a piezoelectric electromagnetic hybrid energy harvester (HEH) based on a planetary gear system, which combined piezoelectric and electromagnetic effects and was mainly used for collecting low-frequency rotational energy. The HEH had a compact structure with dimensions of 140 × 95 mm and contained four sets of piezoelectric energy harvesters (PEH) and electromagnetic energy harvesters (EMH) inside. Through the planetary gear system inside the device, the device could amplify the external excitation frequency, thereby improving energy output efficiency. To verify the effectiveness of the design, an experimental research device was constructed, and external rotational excitation conditions were simulated using motors. The influence of different numbers of magnets and external excitation frequencies on the output performance of the HEH was studied. The results indicated that the HEH could generate an output voltage of 10.4 V and an average output power of 250 mW under eight magnets and an external excitation frequency of 7 Hz. In actual power supply testing, the HEH successfully lit up 60 LEDs, fully verifying its potential for application in low-frequency environments.

## Figures and Tables

**Table 1:** Dimension parameters of the HEH.

Description	Value
Dimensions of the PZT	40 × 10 × 0.2 mm^3^
Dimensions of the cantilever	60 × 10 × 0.2 mm^3^
Dimensions of the square magnet	10 × 10 × 3 mm^3^
Dimensions of the circular magnet	5^2^π × 2 mm^3^
Dimensions of the cover	57^2^π × 8 mm^3^
Dimensions of the pedestal	70^2^π × 8 mm^3^
Inner, outer, and height of the coil	12 mm, 20 mm, 15 mm
Wire diameters of the coil	0.35 mm
Turns of the coil	432
Impedance of the coil	4Ω
Module of the sun gear and planet gear	1.5
Number of teeth of the sun gear	20

**Table 2:** Material parameters of the HEH.

Description	Material
PZT	PZT-5H
Cantilever	301 stainless steel
Square magnet	NdFeB 35
Circular magnet	NdFeB 52
Coil	Copper

## Data Availability

The original contributions presented in the study are included in the article.
